# How Common did the Chronic Schizophrenic Inpatients Suffer from Constipation, and Why? A Cross-sectional Study in A Psychiatric Hospital in Taiwan

**DOI:** 10.1192/j.eurpsy.2025.2265

**Published:** 2025-08-26

**Authors:** Y.-J. Shih, N.-Z. Chen, Y.-L. Chen, H.-W. Chien, Y.-J. Yang

**Affiliations:** 1 Department of Nursing, Tsao-Tun Psychiatric Centre, Tsao-Tun Township, Nan-Tou County; 2 Department of Healthcare Administration, Asia University, Taichung City; 3 Department of General Psychiatry, Tsao-Tun Psychiatric Centre, Tsao-Tun Township, Nan-Tou County, Taiwan, Province of China

## Abstract

**Introduction:**

Patients with schizophrenia are at highs risk for constipation and are more likely experienced severe health consequences than the healthy general population. Due to the heterogeneous definition and the health professionals’ long-term attitude of taking condition for granted, constipation has been neglected, and the relevant evidence is insufficient.

**Objectives:**

This study aims to investigate the prevalence rate of the chronic schizophrenic inpatients, and to address the relevant factors that may be related with their constipation.

**Methods:**

The study adopted the cross-sectional study design with the approach of purposeful sampling. Hospitalised patients with chronic schizophrenia in a psychiatric hospital in central Taiwan were enrolled through the advertising posters, and a total of 300 persons were finally included after screening with inclusion/exclusion criteria. Both subjective and objective data were collected by questionnaires which were developed and performed by the research team, and descriptive and inferential statistical analyses were performed with SPSS, version 25. Statistical significance was set at the 5% level.

**Results:**

The definition of constipation was defined as receiving any laxative agent during the study period, and the point prevalence rate of constipation in the chronic schizophrenic inpatients was 74.7%. Three factors were found relevant with constipation with statistical significance. The combined use of first-generation and second-generation antipsychotic medications (OR=3.28 95% CI:1.14-9.46) was regarded as detrimental factors while both increased education years (OR=0.92, 95% CI: 0.87-0.97) and more exercises (more attendance to the twice-a-day self-initiated aerobic exercises) (OR=0.47, 95% CI: 0.23-0.97) were found protective.

**Conclusions:**

The point prevalence rate of constipation was much higher than other similar studies locally or internationally, but, however, such result also faithfully revealed the fact that constipation is literally a ubiquitous health problem among the chronic schizophrenic inpatients. The researchers suggested that performing health education on constipation, enhancing the extent of aerobic exercises and promoting health behaviours for positive cycle, and discussing with the prescribing physicians to simplify the use of antipsychotic agents may mitigate the risk of constipation.

**Disclosure of Interest:**

None Declared

